# Patients, payers and developers of Orphan Medicinal Products: lessons learned from 10 years’ multi-stakeholder dialogue on improving access in Europe via MoCA

**DOI:** 10.1186/s13023-023-02774-7

**Published:** 2023-06-12

**Authors:** Maria Cavaller-Bellaubi, Wills Hughes-Wilson, Šárka Kubinová, Marc Van de Casteele, Evert Jan Van Lente, Emanuele Degortes, Johan Pontén, Hans-Georg Eichler, Yann Le Cam, Simone Boselli, Anna Bucsics

**Affiliations:** 1 EURORDIS – Rare Diseases Europe, Paris, France; 2 Mereo BioPharma Plc, London, United Kingdom; 3 State Institute for Drug Control, Prague, Czech Republic; 4 Rijksinstituut Voor Ziekte- en Invaliditeitsverzekering (RIZIV-INAMI), Brussels, Belgium; 5 Menarini Group, Florence, Italy; 6 Medicine Evaluation Committee (MEDEV), Brussels, Belgium; 7 Association of Austrian Social Insurances, Vienna, Austria; 8 MoCA Steering Committee Members, Brussels, Belgium

**Keywords:** Access to medicines, Orphan medicinal products, Multi-stakeholder, Early dialogues, Value evaluation

## Abstract

**Background:**

The Mechanism of Coordinated Access to Orphan Medicinal Products (MoCA) was established in 2013 with the intention of developing a coordinated mechanism between volunteering EU stakeholders and developers of Orphan Medicinal Products (OMPs) to support the exchange of information aimed at enabling informed decisions on pricing and reimbursement at Member State level and to evaluate the value of an OMP based on a Transparent Value Framework. The objective of the collaborative approach was to support more equitable access to authorised therapies for people living with rare diseases, rational prices for payers and more predictable market conditions for OMP developers. Over the past 10 years, the MoCA has conducted a series of pilot projects, examining a variety of different products and technologies at different stages of development; and with contributions from a variety of patient representatives, participation from EU payers from a range of Member States and, recently, with EUnetHTA members and the European Medicines Agency participating in the meetings as observers.

**Results:**

10 years on from the establishment of the MoCA, the European landscape has significantly evolved, not only in the field of drug development with increasingly transformative therapies based on novel technologies, but also in terms of larger numbers of approved treatments, increased budget impact and the resulting associated uncertainties; as well as in terms of stakeholder collaboration and interactions. The value of early dialogue with OMP developers, including the EU payer community via their national decision-making authorities, is a key element within this early interaction and contributes to identifying, managing and reducing uncertainties allowing a prospectively planned approach earlier in development and, consequently, to support more timely, sustainable and equitable access to new OMPs, particularly where there is a high unmet medical need.

**Conclusions:**

The voluntary, informal nature of the MoCA interactions creates a flexible framework for non-binding dialogue. A forum for such interactions is needed to achieve the aims of the MoCA and both to support healthcare systems in planning as well as to underpin timely, equitable and sustainable access to new therapies for patients with rare diseases within the EU.

## Introduction

Orphan Medicinal Products (OMPs) are medicines with a marketing authorisation (MA) through the European Medicines Agency (EMA) Centralised Procedure, with the objective of supporting equitable access to needed therapies for rare diseases within the European Union (EU). Despite the contribution of the EU Regulation on Orphan Medicinal Products [1] over the past two decades and the effective overall EU policy framework to support the development and authorisation of OMPs and new treatments for people living with rare diseases, delays and disparities in accessing therapies for rare disease patients are still being reported [2].

Health Technology Assessment (HTA) bodies and Regulators have recognised the importance of early and cross-functional engagement in a unique forum including patients’ representatives and payers with a shared goal of improving timely and equitable access to authorised OMPs in a coordinated manner.

Despite the national competency and responsibility, EU Member States (MS) share the principles of equity and solidarity; face common challenges in providing diagnosis, treatment, and care for patients; and share similar financial and organisational burdens in organising access to novel and often costly therapies. All of these challenges are rendered more acute in the case of rare diseases and OMPs, where small numbers of patients are concerned, where expertise is scarce and scattered, and where potential therapies to address the unmet medical need are increasingly based on emerging technologies and may engender significant economic considerations, including uncommon or new dosing regimens, as is often the case with cell- and gene-therapies.

The Mechanism of Coordinated Access to Orphan Medicinal Products (MoCA) was established as part of the “Process on Corporate Responsibility in the Field of Pharmaceuticals” [3] launched by the European Commission (EC) in September 2010. The Process on Corporate Responsibility consisted of three pillars: (1) Transparency and ethics in the sector; (2) Access to medicines in Africa; and (3) Access to Medicines in Europe, notably in the context of national pricing and reimbursement (P&R) decisions that ultimately determine the real availability of novel therapies to patients. The third pillar was sub-divided into different working groups, one of which focused on access to OMPs within the EU. This was designated as the “Mechanism of Coordinated Access to Orphan Medicinal Products” (MoCA) and was tasked with examining the potential contribution of increased collaboration between EU MS, decision-makers, and stakeholders in supporting the timely, sustainable, and equitable access to novel OMPs and treatments for rare diseases within the EU.

In the 2013 conclusions and reporting from the Process on Corporate Responsibility, the main MoCA outcome consisted of recommendations to develop a coordinated mechanism between volunteering EU MS and developers of OMPs to examine and evaluate the potential value of an OMP, based on a collaboratively developed Transparent Value Framework (TVF). This framework was designed to exchange information in a structured format by enabling informed and data-driven decisions on P&R at MS level. The participants believed that such a structured dialogue, ultimately, would lead to more rational prices for payers, more predictable market conditions for industry and more equitable access for patients [4].

The TVF, as originally developed, lists the elements which are important criteria contributing to the value of a new OMP: (1) Are there available alternatives/degree of unmet need (including non-pharmaceutical treatment options); (2) The relative effectiveness/degree of net benefit relative to alternatives (including no treatment); (3) Incremental/major/curative response rate (based on best available clinically relevant criteria); and (4) Degree of certainty (well-documented). Specifically, the TVF is an instrument for supporting increased transparency of the relationship between value and pricing by defining the criteria of value to payers in a qualitative and semiquantitative manner. The TVF is based on the consensus between various stakeholders in the Working Group of the Process on Corporate Responsibility on how to assign value in a consistent way and, as such, was considered a relevant framework to support collaborative dialogue by providing a structure in a consistent way, to ensure equal treatment of patients and providers across the wide range of OMPs and technologies [4]. New OMPs could be assessed according to how well they fulfilled the different criteria at a given point in time (Table 1).

**Table 1:** Adapted from the Transparent Value Framework [4]

Criterion	Degree of fulfilment		
	Lower degree	Medium degree	High degree
Are there available alternatives, including non-pharmaceutical treatment options (unmet need)	Yes, new medicine does not address unmet need	Yes, but major unmet need still remains	No alternatives, except best supportive care—new drug addresses major unmet need
(Relative) effectiveness, degree of net benefit (clinical improvement, quality of life, etc. vs. side effects, societal impact, etc.) relative to alternatives, including no treatment	Incremental	Major	Curative
Response rate (based on best available clinically relevant criteria)	< 30%	30–60%	> 60%
Degree of certainty (documentation)	Promising but not well-documented	Plausible	Unequivocal

Subsequently, key members from the MoCA Working Group agreed to further pursue discussions to explore operationalising the recommendations in MoCA pilot projects discussing real OMPs in development. These key members consisted of the Medicines Evaluation Committee (MEDEV) [5], which is a working group of experts from statutory health insurance institutions and HTA bodies in Europe and EURORDIS-Rare Diseases Europe, a non-profit alliance of rare diseases patient organisations [6]. Following the establishment of the first pilots, the MoCA published its revised terms of reference in 2016 [7]. This document clarified the procedure of the MoCA pilots and led to the establishment of a MoCA Steering Committee. EURORDIS agreed to host the MoCA webpage [8] which provides the information for potential applicants. In 2018, the EMA and EUnetHTA start participating as observers in the pilot projects.

The dialogue in MoCA over the past decade extended beyond the initial framework of analysing support for decisions on P&R, which remain the exclusive competence of each EU MS. The MoCA Steering Committee, together with participating payers, patient community and company representatives has reviewed the experience gained from the pilots to capture and evaluate the benefits for different stakeholders in terms of facilitating the shared goal of timely, equitable and sustainable access to OMPs within the EU provided by the early collaborative dialogue in MoCA.

## Methods

A MoCA pilot concerns a specific OMP or potential OMP, or a group of related OMPs. It is initiated by the developer, usually a pharmaceutical company, by contacting a member of the MoCA Steering Committee. The Steering Committee then assesses the suitability of the OMP(s) for a MoCA pilot, based whether there is a necessity for discussions on optimizing access to the OMPs. However, OMPs can be topics of a MoCA pilot in every stage of the life cycle.

If the product is deemed suitable, potential participants from MEDEV members are contacted. MEDEV volunteers represent the payers. Under the payers’ employment contracts, the MoCA discussions are conducted subject to the same levels of confidentiality that would be the case in a regular “one on one” engagement with a national P&R authority.

EURORDIS manages the process for involving the patient representatives at all stages of the MoCA dialogue engagement, from the first identification of the participants best suited to cover the perspectives on the specific rare disease in question to be addressed in the meetings; and actively mentors them through the process. All patient representatives involved in these meetings sign a declaration of interests and a confidentiality agreement to ensure transparency and comply with the MoCA internal procedures. EURORDIS representatives also participate as members of the Steering Committee. Other institutions, such as the EMA and EUnetHTA are contacted, and they may participate as observers. In this way, MoCA provides a unique multi-stakeholder setting because all the stakeholders are represented: patients, payers, company representatives, regulators and HTAs.

**Fig. 1:**
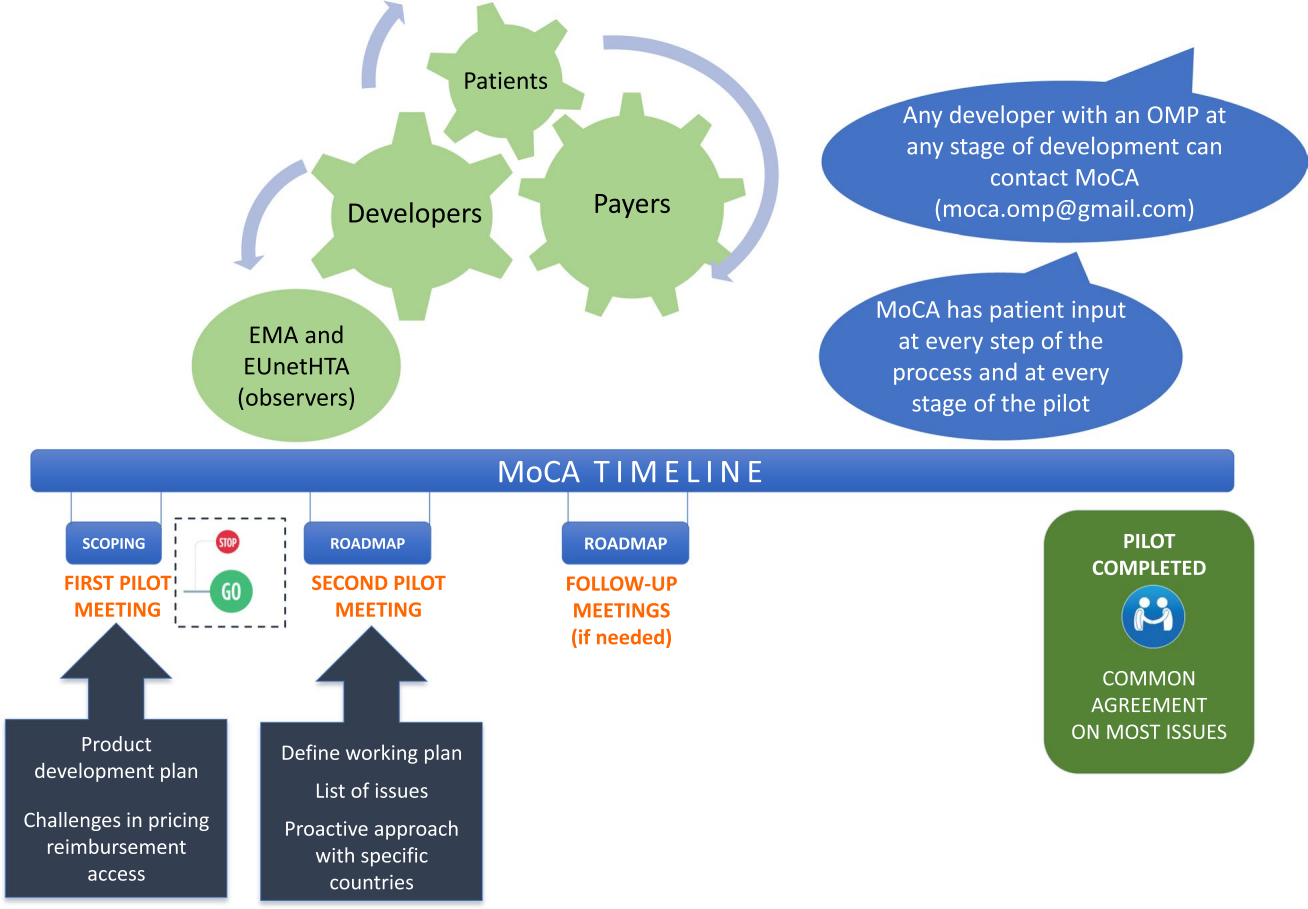
MoCA processes, timelines and deliverables

Participation in the discussions is confidential, voluntary, non-binding, and free of costs for developers. More than one meeting may be held per OMP. In the first meeting, the developer is expected to present a timeline of the further development of the product and a list of topics to be discussed. The OMP developer drafts minutes of the meeting that are shared with participants to support continuity in case of follow-up engagements. Patients’ contributions deal not only with explaining the challenges they experience and how the product in question can address those; but they can also attempt to bridge the gap between payers’ and developers’ views. The aim of these discussions is to find some consensus on the issues discussed; while acknowledging that this might not always be achievable (Fig. 1).

Initially, MoCA meetings were held in person following the regularly scheduled MEDEV meetings. During the COVID-19 global pandemic, the MoCA meetings shifted to an online platform, which brought unanticipated advantages for all stakeholders by facilitating broader participation on a more consistent basis. For the volunteering rare disease patient representatives, who previously were not always able to participate in face-to-face meetings due to issues with travelling and/or other time commitments; for the payers, because they could contact experts who were not foreseen to be participating in the MEDEV meetings; and for the developers because the online meeting format reduced travel costs and allowed participation of a broader representation of expertise directly involved in the clinical development programme.

In 2017, all participants in MoCA meetings were contacted and invited to participate in an anonymized online survey [9]. In addition, further developers who were thought to be involved in OMP development at that time were also invited to participate. The purpose of this survey was: 1) To ascertain the usefulness of MoCA to the involved stakeholders; 2) To understand potential barriers to being involved in the MoCA dialogue; and 3) To elicit suggestions on how to improve MoCA. The results of the survey were discussed at a meeting of the EMA with the payer community in 2017 [10] and the results of this discussion are presented below.

## Results

Since MoCA was established, 47 meetings have taken place. In total, MoCA has accommodated 16 developers and discussed 23 different OMPs (Table 2) along different stages of the development (Table 3) and Anatomical Therapeutic Chemical (ATC) code classification (Table 4). 20 patients’ representatives have participated, some of them participated in more than one meeting. 12 of the products discussed were small molecules, five biologicals and five advanced therapies, and one therapeutic approach was classified as “other”.

**Table 2:** MoCA meetings and participation (09/2013 – 12/2022)

Total number of meetings	47
Meetings per developer	Average: 2.9/ Median: 2/ Maximum: 9
Meetings per OMP	Average: 2.04/ Maximum: 6
Number of OMP developers participating /consortia	16
Number of OMPs discussed	23
Number of patients representatives	20
Number of payer-representing institutions that attended at least 1 meeting	27
Other participating institutions: Academia, as well as EMA and EUnetHTA as observers

**Table 3:** Development stage of the OMPs discussed in MoCA meetings

Status at first contact	Number	Status as of 20 Feb. 2022
Pre- clinical stage	3	2 in development, 1 terminated
Phase 1 or combined Phase 1 and 2	4	1 in Phase 3, 1 authorised, 2 terminated
Phase 2	4	1 authorised, 1 in Phase 3, 2 terminated
Phase 3	7	3 authorised, 1 under evaluation by EMA, 2 in Phase 3, 1 terminated
Marketing authorisation application submitted	2	Both authorised
Already authorised in the EU	3	1 withdrawn, 1 off-patent, 1 marketed but not orphan

**Table 4:** The Anatomical Therapeutic Chemical code (ATC) classification of OMPs discussed in MoCA meetings

ATC code	Number of OMPs
A(limentary)	5
B(lood)	4
C(ardiovascular)	3
(Onco)L(ogy)	2
M(usculoskeletal)	3
N(ervous system)	3
R(espiratory)	2
S(ensory)	1

The topics discussed during the meetings include, amongst others, the epidemiology of the condition in question and the definition of the patient population most likely to benefit; the relevance of endpoints selected by the regulators and the demonstration of potential added value by the product; the needed data requirements prior to initial reimbursement as well as post-authorisation and how and when these can be collected; and potential novel payment models. The authors recognise that discussions around specific pricing are very sensitive for all stakeholders involved, and the MoCA is not intended to negotiate prices. However, the MoCA offers an opportunity to discuss potential parameters for managed entry agreements.

### Statistics

#### Benefits for participating stakeholders

Stakeholders have highlighted the benefits of participating in MoCA (Table 5) because of its nature as a unique forum where all stakeholders are present, as mentioned above. Patient engagement in multi-stakeholder dialogue is essential, particularly in the field of rare diseases where expertise is scarce and scattered and, very often, the people living with a rare disease are the best source of such expertise in their specific condition. The multi-stakeholder exchanges incorporating all decision-makers are a valuable opportunity to not only improve awareness of the diseases, diagnostics and treatments; but also to identify and discuss potential barriers to access; to increase affordability and to improve cost-effectiveness by improving the evidentiary base; and to develop innovative and practicable payment models to support actual access to new therapies in a timely and sustainable manner, often against a backdrop of lack of existing therapeutic alternatives.

**Table 5:** Summary of potential and realised benefits of MoCA as a multi-stakeholder approach in Europe

Developers	Regulators	HTA	Payers	Patients
Payers input early enough to support a clinical development programme adapted for downstream decision-making	Insight into potential “downstream” problems impacting anticipated availability, opportunity for direct interaction with HTAs and payers	Enhanced awareness of the condition and the actual unmet medical need to support potential future comparative assessments	Better budget impact awareness and predictability; insights into potential treatment-eligible population(s), epidemiological data; opportunity for early understanding of treatment setting or pathways (e.g., hospital vs. outpatients setting, first or later line therapy…)	Quicker and broader availability of an individual OMP and increased equity across EU MS
Increased predictability and planning for launch timing, likely uptake and barriers	More efficient Scientific Advice (if advice is taken into account)	Better data for HTA (if advice is taken into account)	Unique platform for multi-stakeholder and multi-country engagement on an individual OMP; opportunity to discuss potential parameters for managed entry agreements	Better understanding of needs and expectations from decision-makers in determining access and availability questions
Clear understanding from all data-customers about data-requirements to support value demonstration, initial P&R decisions and maintenance	Input into consolidated post-authorisation data-collection in conjunction with other data-customers – avoiding fragmentation and supporting inclusion of EMA tools [19] including Guidance and link with EUnetHTA / HTA tools, e.g., REQqueST [20]	Dialogue that includes the payer / budget-holder perspective on a given OMP, especially for countries where the HTA / payer agencies are split	Sharing of expertise with different MS / transparent understanding of clinical value, natural history, and related organisational aspects	Ability to inform and engage with OMP developers to secure relevant endpoints, expectations and needs are included
Guidance on prospectively designed post-marketing evidence generation / data-gathering / requirements based on gap identification from all data customers from earliest phases of development / market launch planning	Secure HTAs and Payers are aware of the regulatory rationale for decisions on endpoints and their relevance to the therapeutic area in question			Better, coordinated follow up and collection of PROs and real-life experience

##### Patient representatives

For the patients, MoCA meetings are a “safe space” to share their experience of living with the condition under discussion, to better understand the potential contribution of new therapies and to outline their expectations towards the new treatments. During these meetings, better and coordinated follow-up and discussion of patient reported outcomes (PROs) and real-life experiences helps developers of OMPs to tailor the clinical trial design.

Having patient representatives participating in the meeting and providing their real-life experience of living with the condition can also be a significant added value because the discussion of unmet need can be more holistic and can encompass real-life experience of the impact of a given rare disease. Patient representatives participating in MoCA meetings have highlighted the value of the early dialogues, one participant commenting that “cooperation with all stakeholders must be more intensive and this kind of meeting must be held on a regular basis”.

For the payers, regulators, and developers MoCA provides an opportunity to hear from patients and their needs.

##### Payers

For the payers, MoCA is an opportunity to prepare in advance more informed decisions on treatments for diseases about which not much may be known. Within one setting, valuable discussions can be held on a range of different elements that have a direct impact on national healthcare decision-making, including patient (sub)populations, posology, alternative treatment options, possible budget impact, organisational aspects and cross-border care in the context of a given rare disease. Payers are better able to assess how far the new technology addresses real unmet medical needs from the direct engagement of patients living with the disease in question. The potential exists to speed up reimbursement decisions when payers’ evidence requirements are met. MoCA also offers an opportunity to prepare for the post-launch evidence generation plan, in cases where a MA has been or is expected to be granted on the basis of a limited level of evidence, which is often the case for OMPs. Such authorisations increase uncertainty on the value of the new technology as compared to alternatives (if existing) and, as such, having a prospectively designed plan to develop evidence to support P&R decisions on a longer term is an important element for healthcare systems.

Another key outcome is the trust and mutual understanding between payers and the OMPs developers through not only the MoCA dialogue itself but also afterwards in the actual national P&R processes. Early dialogue allows a more prospective, rather than reactive approach; and creates the possibility to anticipate potential requests that would otherwise not been expected. The early opportunity to address payers’ needs is one of the key elements of added value provided by MoCA because in other settings, e.g., the EMA-EUnetHTA dialogues [18], payers are not included, which can result in additional and unanticipated requirements are requested by payers at a later stage.

##### OMP developers

For OMP developers, the MoCA pilots have provided numerous benefits at all stages of development. In early phases, the benefits include the possibility to gain early payer input on a clinical trial programme and the potential resulting reimbursement dossier and, in addition, to be able to explain the unmet need for conditions that could be less known to payers. The MoCA process helps to clarify questions early on in the development programme, e.g., acceptance of endpoints, expected comparators, perceptions of conditional marketing authorisation, evidence generation both pre- and post-authorisation; and has helped guide strategies to address data gaps, e.g., through a registry strategy. The TVF, while providing criteria for evaluation at a high level, creates a structured format for discussing the scientific value of a novel therapy and its potential place in healthcare systems.

Having the opportunity to explore the epidemiology and heterogeneity of a given condition and potential subsets of severity, together with the willingness to treat, and the expected treatment outcomes are critical elements in a future request for coverage. Reviewing the Target Product Profile and the eventual anticipated label at an early stage in a multi-stakeholder setting is a critical element around understanding potential patient numbers and forecasting, both for developers and healthcare systems. Further, where rare diseases have had no therapy to date and might, therefore, be “invisible” to the pharmaceutical budget holders, allowing sufficient time to explore clinical benefits and impacts of a potential new therapy is important.

In addition, the MoCA platform provides a unique opportunity for OMP developers to hear feedback from a group of ‘real payers’. MoCA provides access to a group of decision-makers actively involved in the assessment and decision-making for novel therapies. Traditional platforms such as Payer Advisory Boards, which tend to include proxy or former payers, have an important role to play; but at the same time these differ from MoCA because while having extensive experience, might lack the latest nuances of a given country authority. The involvement of current payers in MoCA, as opposed to retired or “ex-payers” typically participating in Advisory Boards, means that the payers participating are likely to be fully engaged with a dossier of an OMP of current or future relevance and will be aware of the current sensitivities, rules or areas of particular attention for their country payer authority.

Having the patient perspective included at the same table with the payers and other decision-makers allows for a more impactful description of the challenges of living with a given rare disease – something which a developer alone would struggle to be able to portray accurately and, in a manner, relevant to the national decision-makers. Some OMP developers observed that it was “only when payers heard the perspective of the patient representative did, they really understand the challenges in the current standards of care and the value that a novel therapy could bring”.

Discussing the clinical trial protocols to better understand perceived data gaps by payers has also helped a number of companies develop a registry or evidence-generation strategy early on to address these gaps. Having an early dialogue can encourage all stakeholders to come closer together on their position and to identify data gaps early on to accelerate negotiations once these become live. Post-launch data collection requirements are currently progressed with each individual data customer individually.

Using the TVF as the basis to achieve a “common language” has also proven a valuable exercise that can facilitate individual company decisions. For example, one company shared that, “following the discussion with MoCA we made a concession on price in order to ensure faster access to the treatment across Europe. At the same time, the payers in the room had a better understanding of the value of the therapy and we believe this helped with negotiations in a number of countries”. The trust built up by transparent dialogue within the MoCA about a specific OMP or programme can also support pricing discussions on other, different, products in the company portfolio outside the MoCA process because the company is known to the payer authorities and a platform for open engagement has been established.

Some elements could make the MoCA even more productive for companies seeking to launch OMPs in the EU. The main challenges companies face in participating in MoCA relate mainly to the inconsistency in terms of payers’ participation and discussion at the different meetings. As a voluntary initiative, it is not to be expected that all payers from the 27 EU MS participate in all MoCA meetings. However, the uncertainty about the number of participants can be a deterrent for companies, which invest significantly in the preparation of the discussions. Some companies participated in MoCA meetings with more than ten country payer representatives, while others only had two. The online format of meetings following the COVID pandemic has contributed to increasing the consistency levels of participation. Having a minimum threshold of payer participants for the MoCA meeting to go ahead would support consistent engagement by developers of OMPs. Similarly, having human resources to secure a full-time expert who can manage the agenda and encourage participation by MS authorities would be useful. Having neutral expert support, i.e., not the company concerned, in highlighting to the participating country payer authorities if there is an aspect of particular relevance for them about the programme or OMP under discussion, e.g., an elevated genetic prevalence in their country, the presence of a specific centre of expertise, national policy or other reasons why the OMP could be particularly relevant to their country and thus, their work, would be an added value to allow effective evaluation and decisions about participation from MS’ authorities.

##### Regulators and HTA bodies

Pricing and reimbursement issues are not the competence of EMA [11]. However, the definition and optimisation of treatment-eligible population(s), accurate description of expected clinical benefits and potential harms, identification of knowledge gaps, and ensuring that relevant knowledge gaps are closed by post-authorisation evidence generation, are core responsibilities of the EMA – as is communication of these issues to external stakeholders. The EMA has rightly understood that HTA bodies and payers are among the most interested, knowledgeable (and sometimes most critical) “customers” of their decisions and communications. Therefore, the EMA has been interested in participating, supporting and exploring dialogues with and between payers, HTA and companies developing OMPs before a potential MA is granted, for example in the context of the EMA/payers meetings [10, 12, 13].

The MoCA provides a voluntary forum for the EMA to understand the requirements from payers and HTA-bodies at early stage, which can be integrated in the early dialogues of the regulator in early advice on the design of Phase 3 clinical studies. The EMA will also be able to explain to national payers and/or HTA bodies the reasons why the new product might be assessed to have a positive benefit-risk-ratio. These considerations can be taken into account in the assessments of the HTAs and payers when reflecting the additional benefit to the current standard of care of the new OMP. Further alignment on post-launch evidence generation to maximise available data to support decision-making on safety, efficacy and effectiveness will be an important opportunity, particularly in the field of rare diseases and OMPs where patient numbers are limited and where cross-function and cross-border EU collaboration can add value at an EU but also potentially at a global level. Therefore, EMA’s participation in MoCA as observers is well justified and valuable.

## Discussion

### 10 years of experience of MoCA

During the 10 years since the establishment of MoCA, the medicine development landscape has significantly evolved (e.g., with the introduction of gene-, cell- and other transformative therapies) and, in addition, so have the corresponding challenges in evaluating potential therapies and reaching decisions on reimbursed access.

The EMA has not only encouraged and supported Scientific Advice [14] it has also established increasingly structured and formalised dialogues with EUnetHTA including, most recently, the Joint Scientific Consultation (JSC) pilot projects announced in 2021 and having a second round in 2022–2023 [15]; and also with the payer community under the EMA and Payer Community meetings initiated in 2017 [10], the second meeting in 2019 [12] and the most recent in 2021 [13]. This has allowed exchanges between a subset of stakeholders. However, the MoCA is the only platform that brings all participants together at the same time and around the same questions, which allows a full exchange of opinions addressing all the relevant points in one setting.

Individual country frameworks are evolving to accommodate more inclusive evaluations being HTA or P&R discussions. Also, an increasing number of MS payer authorities are evaluating the benefits of cross-border collaboration further downstream, including the BeNeLux-I-A [16] and FINOSE [17], amongst others. The value of collaborative dialogue is, therefore, acknowledged by a broad range of stakeholders within the evaluation and reimbursement of novel therapies. MoCA uniquely offers such collaboration with all stakeholders from earlier stages of development, allowing feedback to be included both in a potential clinical development programme and post-authorisation activities.

Importantly, the Regulation on Health Technology Assessment [18] (“the HTA Regulation”) was adopted in December 2021 and introduces a permanent legal framework for joint HTA at European level. The Regulation aims to provide for a mechanism that ensures that any information, data, analyses, and other evidence required for the Joint Clinical Assessment (JCA) should be submitted only once at the EU level by the health technology developer.

It will cover JCA as well as JSC with health technology developers to provide consultations in particular in concerning all relevant clinical study design aspects, or clinical investigation design aspects, including comparators, interventions, health outcomes and patient populations.

While the HTA Regulation for medicinal products with new active substances for oncological indications and advanced therapy medicinal products (i.e., gene, tissue, and cell therapy products) will be implemented from 12 January 2025, the HTA Regulation’s application will extend to OMPs from 13 January 2028 and from 2030 to all centrally authorized medicinal products.

These changes may well affect the work of MoCA, making it useful as a low-threshold, informal “entry point” for developers to discuss their products at an earlier stage on a multi-stakeholder platform, and to coordinate their interactions with agencies and various competent authorities, with patient input.

### Remaining challenges

During the 10 years since the establishment of the MoCA, pharmaceutical developments have created an increasingly complex environment with high uncertainties and with often high prices in an environment of healthcare budget constraints and the global COVID-19 pandemic. These have highlighted the need for early dialogues with all stakeholders involved in decision-making that potentially could help to solve some of these issues. MoCA multi-stakeholder exchanges are an opportunity to identify and discuss potential barriers to access and to discuss the expected added benefit to a comparator and, if significant uncertainties are identified, MoCA provides a forum where payers can reflect how these uncertainties can be addressed in payment models.

MoCA is not an opportunity to “pitch the product”; and is not a “cure-all”, but it is, at a minimum, an opportunity to frame the questions and to support an early understanding of downstream needs from decision-makers via a flexible, multi-stakeholder platform, that is independent, informal and free of charge, playing a key role in coordinating the input from key stakeholders. The informal aspect has been key to translate the MoCA learnings into national reimbursement processes for each EU MS separately, respecting different national and agency competences, and has underpinned engagement downstream as well as allowing input from upstream.

Based on the learnings from the past decade, the authors are aware that financial and/or human resources would be needed for further engagement, to support more structured dialogues, while keeping MoCA’s flexibility and independence. Rare diseases patient representatives need to be involved beyond the time of MA. To improve the efficiency of MoCA, better alignment with other processes, such as Scientific Advice [14] and the EMA-EUnetHTA consolidated Joint Scientific Consultations [15] and the EMA-Payer Community forum [10, 12, 13] is needed, particularly on key questions of evaluation and assessment. Payer participation should be encouraged and supported by national authorities.

For developers of OMPs, more guidance on when, where and how to approach MoCA it will be of benefit. Many developers, particularly Small- & Medium-sized Enterprises (SMEs) might find it challenging to differentiate between MoCA and the variety of existing initiatives – from EMA’s Scientific Advice [14], with or without HTA input, to the dialogue with regional consortia, such as BeNeLux-I-A [16] and FINOSE [17]. Highlighting the informal and inclusive nature of MoCA should be a priority.

### Next steps: strategic learnings for the future of MoCA?

In order to secure the sustainability of MoCA beyond the individuals who have been leading and coordinating the voluntary platform, the authors believe that now is the time to establish a more solid organisational footing for MoCA and its meaningful work in the longer term. Given that MoCA supports the EU goal of equitable access to novel therapies, the authors believe that an EU Joint Action would be an appropriate vehicle to provide funding in order to build on the work conducted over the past 10 years as well as to further develop a robust and sustainable multi-stakeholder platform for early dialogue as discussed in the second EMA-payer community meeting in 2019 [12].

MoCA in a broader and more structured form could support the negotiation of fair and equitable prices on a European scale in exchange for an earlier access to patients across Europe. This could be achieved via multi-year buying commitments and revenue predictability for developers of OMPs. MoCA discussions could also facilitate a coordinated, European plan based on input from all decision-makers, which could address the uncertainties at the time of MA for many OMPs by providing a plan to develop additional evidence to reassess the value of a product at an agreed time point.

Future involvement of the European Reference Networks, the continued involvement of the EMA and EUnetHTA as key stakeholders, in addition to the payers and the patient community, all of them with a stronger mandate to align on key questions of evaluation and assessment would ensure effective, timely, broader and meaningful input from all implicated stakeholders; and would, at the same time, contribute significantly to speeding up patient access to needed therapies while supporting increased equity across European MS. Learnings from this experience could also be of value to the international rare disease community beyond the EU borders.

## Conclusions

MoCA is a unique platform providing informal and confidential but structured early dialogue between all stakeholders and, as such, offers the potential to become a permanent structure; and a potential solution for the accessibility, availability and affordability challenges that have been identified in the field of OMPs and rare diseases.

Having a multi-stakeholder framework to bring together what could otherwise be fragmented data collection exercises has the potential to enhance the quantity, quality, and relevance of gathered data. Utilising the opportunities provided by the EU-level collaboration could create knowledge relevant to informing understanding of given rare conditions in other jurisdictions and geographies.

On the basis of the 10 years of valuable experience generated by the MoCA pilots, the authors believe that it is now time to move to the establishment of a permanently supported platform. This would save and optimise payers’ resources as well as allowing for a structured collaboration between competent authorities to assess the value of OMPs.

## Data Availability

The datasets generated and/or analysed during the current study are not publicly available due to the confidential nature of the MoCA discussions.

## Abbreviations

ATC: Anatomical Therapeutic Chemical
EC: European Commission
EMA: European Medicines Agency
ERNs: European Reference Networks
EU: European Union
HTA: Health Technology Assessment
JCA: Joint Clinical Assessment
JSC: Joint Scientific Consultations
MA: Marketing Authorisation
MoCA: Mechanism of Coordinated Access
MEDEV: Medicines Evaluation Committee
MS: Member State
OMPs: Orphan Medicinal Products
PROs: Patient Reported Outcomes
P&R: Pricing and Reimbursement
SMEs: Small- & Medium-sized Enterprises
TVF: Transparent Value Framework
